# Pharmaceutical entanglements: an analysis of the multiple determinants of ADHD medication effects in a Chilean school

**DOI:** 10.1080/17482631.2017.1298268

**Published:** 2017-05-22

**Authors:** Sebastian Rojas Navarro, Scott Vrecko

**Affiliations:** ^a^ Department of Global Health and Social Medicine, King’s College London, London, UK; ^b^ Facultad de Psicologia, Universidad Diego Portales, Santiago, Chile

**Keywords:** Stimulant medication, ADHD, medicalization, pharmaceutical entanglements, children, ethnography, constraints, agency

## Abstract

This article draws upon findings from ethnographic fieldwork conducted in a Chilean school to explore how the effects of globally circulating ADHD medications emerge within the localized contexts of everyday users. An analysis of observations of children on ADHD medications within classroom settings is developed which challenges the assumption, pervasive within biomedical paradigms, that the effects of such medications can be understood as resulting directly from their chemical properties and biological modes of action. Our case study highlights the significance of multiple, interacting determinants of drug effects in an everyday setting, focusing in particular on classroom dynamics, teacher–student relations, and the agency of children taking the medications. We conclude that while ADHD medications may act in part by altering physiological processes, an adequate account of their effects requires that analytic attention extends to the sociomaterial contexts in which medications and users are embedded.

## Medicalization perspectives on ADHD

In 1976, Peter Conrad published a book that has become one of the major milestones for studying children’s mental health and illness from a critical standpoint (Conrad, 1976). In contrast to biomedical paradigms, which assumed that psychiatric disorders reflected biological abnormalities, Conrad suggested that psychiatric diagnoses are, to a significant extent, socially and historically constructed. In one of the first sociological analyses of attention deficit/hyperactivity disorder (ADHD), Conrad argued that the emergence of this diagnostic category was an example of the dynamic and continuous process of “medicalization” (Conrad, 1992, 2005), involving the extension of medical authority across new areas of personal and social life. For Conrad, the treatment of ADHD was best understood in terms of the medicalized social control of children and problematic forms of behaviour.

In work that has continued over decades, Conrad (1975, 1992, 2006) has examined connections between steadily increasing rates of ADHD diagnosis and ADHD medication consumption, the dynamics of pharmaceutical science and industry, and governmental involvement (especially in the USA) in the promotion of medication-based approaches to managing childhood behavioural disorders (Conrad, 1975). As a result of Conrad’s work, along with a vast number of similarly oriented studies, ADHD has become “both the most extensively studied paediatric mental disorder and one of the most controversial” (Mayes, Bagwell, Erkulwater, 2009, p. 1), and psychostimulant treatments have come to be widely considered as tools used by experts and other authorities to exert control over individuals who have difficulties meeting the expectations associated with “normal” childhood behaviour and/or who struggle to adapt to the demands of school environments.

Such scholarship—as well as recent work on biomedicalization (Clarke, Mamo, Fosket, Fishman, & Shim, 2010), and studies of how pharmaceuticals may be put to work within pre-existing forms of social and legal control (Bourgois, 2003; Roberts, 1997; Schoen, 2005; Vrecko, 2010)—has provided many important insights into the relations between ADHD psychiatry, pharmaceutical treatments, and the regulation of deviant or problematic forms of behaviour. The concept of medicalization is now frequently used in both popular and academic discussions that suggest the widespread consumption of ADHD medications among the young (a phenomenon observed to have appeared first in the USA, and subsequently to have spread through much of the rest of the world) represents an oppressive form of social control imposed on children (Conrad, 1979; Harwood, 2005).

While such perspectives constitute an important backdrop for this article, our intention here is not to reproduce the main arguments associated with them. As Nikolas Rose has pointed out, medicalization perspectives have become so firmly established within social science scholarship that their relevance is sometimes taken for granted as a given—to such an extent that medicalization now risks becoming a cliché of social analysis (Rose, 2007). Although we have no doubts of the continued relevance of medicalization theories to ADHD, and this article is in fact motivated by many of the same concerns and questions that are articulated in medicalization literature, we do share some of the concerns expressed by Rose. In particular, we believe that scepticism is warranted towards analyses which suggest that the experiences of millions of children around the world can be understood as fundamentally similar. While increasing rates in the production and consumption of ADHD medication in a variety of national contexts can certainly be taken as indicators of a global phenomenon, we claim that these accounts offer little evidence to support the idea that the manifestations of this phenomenon are not prone to be altered by contextual and sociomaterial elements. In this sense, it becomes difficult to support the idea that this phenomenon manifests itself locally in a uniform manner, regardless of individual circumstances or of wildly divergent geographical, cultural, and socioeconomic contexts.

## Focus and aims of this article

Reflecting a conviction that the particularities of individual bodies, the locations in which they are situated—not to mention the complex interplay between these—may be relevant, even necessary, for deepening understandings of how and why ADHD medications are used, and with what effects, in what follows we examine medications and medicated children as phenomena that are interwoven within complex sociomaterial realities of the everyday world. Conceptualizing these in terms of “pharmaceutical entanglements”, we attend to a set of interactions between ADHD medications, the children taking them, other actors that play a significant role in relation to the fieldwork site (for example, medicated children’s classmates and their teachers, but also non-human elements of classrooms settings) as well as the institutional dynamics and forms of local knowledge that influence (but do not determine) expectations of how and why the medications unfold their effects.

This approach stands in tension with, and even challenges, epistemological positions that are entrenched among the main groups of experts involved in generating authoritative accounts of ADHD medication, including those with clashing views: for example, biomedical practitioners consider psychostimulants to be biology-targeting substances yielding therapeutic effect because of basic chemical properties, while many scholarly critics in the social science and humanities characterize them as instruments of social control. In both cases, it is generally assumed that medications and their effects have a single, true nature that can be discovered. In sharp contrast, we consider the possibility that the same ADHD drug may have multiple realities, depending on how they are enacted as everyday objects within divergent life worlds. This alternative approach is informed by work within the interdisciplinary fields of “Science and Technology Studies” and “Childhood Studies”, particularly the efforts of those attempting to devise analytic frameworks that can account for the simultaneously social and material nature of human life (e.g. Prout, 2005; Rose, 2013), and related issues about how best to understand the nature of human—and non-human—agency (e.g. Pickering, 1995; Prout, 2011). In common with much of this work, we are conscious of the pitfalls of excessive theoretical abstraction and over-generalization, and attempt to avoid them by grounding conceptual discussions in the details of an empirical study—in this case, ethnographic research in a Chilean school.

In what follows, we explore the dynamics that emerge as human and nonhuman agents come together—and in so doing, alter and modify one another—in an everyday setting commonly associated with ADHD medication use among children. Seeking to avoid the pitfalls and shortcomings linked to both socially and biologically reductionist explanations of how ADHD medication works, and why it is used (e.g. sociological accounts claiming that ADHD medication is used as a coercive instrument of social control, and bioscientific understandings which reduce the effects of stimulant medication to mere drug chemistry), we develop an empirically grounded account which highlights the complexity of pharmaceutical entanglements between ADHD medications, the children taking them, and the particularities of the setting in which they do so. This divergent and more nuanced analysis to the topic allows us to understand how the unfolding of certain properties and effects of the medication are highly sensitive to the sociomaterial context in which these elements come together, leading us to considerate the contingent nature of drug properties and effects.

For this purpose, we begin by outlining and briefly introducing our methods and the context in which fieldwork was conducted. We then present three stories collected while doing fieldwork, each of which highlights the complex dynamics in which medicated children, their teachers and classmates, along with nonhuman actors, are involved in the classroom setting. The purpose behind sharing this ethnographic material is to reveal how the effects of stimulant medication do not just happen in a space isolated from contextual determinants. Contrarily, they take place under particular circumstances and in a specific setting where school expectations about the learning process of children, different pedagogic techniques, teachers’ personal views about education, and children’ own interests converge, influencing how the effects of the medication unfold. Our description and analysis of fieldwork materials highlight how children’s consumption of drugs may lead to a variety of different potential outcomes, which are determined by the interacting actions of medications and children on one another. We conclude that both the effects of ADHD drugs, and the circumstances of children taking them, are more complex than is acknowledged in many social analyses.

## Context and methods

Data presented in this article was gathered by the lead author, who conducted ethnographic research in a Chilean educational institution. Over the course of 8 months of fieldwork he spent three full days a week observing children aged 10–11, within four classrooms, each of about 30 children segregated by gender (i.e. there were two classes of boys and two of girls). In addition to observations recorded regularly in fieldnotes, semi-structured interviews were held with medicated and non-medicated children, as well as with school staff such as psychologists, teachers and administrative personal.

In total, over 120 children and 15 staff members participated in research that was reviewed and approved by the researcher’s home institution (King’s College London). Access was granted after 2 months of negotiations with a teacher who was the initial point of contact, and various school authorities who granted the various approvals necessary for the research to proceed. After securing access for the upcoming months, information letters and consent forms were distributed to children, parents, and staff members. Standard procedures for informed written consent were used for adult participants, while children’s participation required agreement from children, as well as written consent from a parent.

Before start conducting interviews, the lead author spent the first 2 months of fieldwork mainly “hanging about” in classrooms (Hammersley & Atkinson, 2007, p. 45). His role in the classes varied according to the desires of the staff members who were teaching them: occasionally teachers would include him in teaching activities as a sort of teaching assistant, or ask him to remain observing from the back of classrooms; usually, however, he was permitted to move about freely and to walk around, talk to children, take notes, or leave the classroom. Children generally reacted to the researcher’s initial presence with interest, as well as some apparent suspicion. However, rapport and trust were established fairly quickly, with students accepting the researcher’s presence and readily engaging in informal conversations about mundane matters such as football, television shows, and music. After a couple of weeks, children also started speaking freely with the researcher about their everyday lives inside the school, including details of their relationships with classmates and teachers, and opinions on various topics, such as whether a teacher’s decision was fair, or whether a class session was fun or boring.

In accordance with institutional ethics requirements, data were only collected in relation to individuals who had consented to participation. In order to ensure anonymity, names of schools and individuals have been replaced with pseudonyms. Therefore, we will refer to the schools for boys and girls as “Mount Sinai” and “Bethlehem”, respectively. Both names have been chosen since they refer to places with symbolic importance to Catholic tradition, in the same fashion that the original names of the school do.

## Pharmaceutical entanglements

It is a warm Friday in April, watching the proceedings of a Technology class, I am once again confronting one of the everyday classroom realities that I had hardly thought about prior to fieldwork, but which now regularly occupied my mind. Namely, noise—or noises, to put it in plural terms that do better justice to the multiplicity of sonic events that come together in classrooms and yield various effects, including continual threats of disruption. While teachers were often successful in their efforts to confront these, they were not always—at times, including mornings like this, noises could rise to the point where it felt the entire classroom might easily slip out of control.

Rosario—the teacher in charge of teaching the Technology class—looks at me. She appears sick and tired of the situation. Looking evidently overwhelmed by what is happening around her, she starts nagging the students in order to decrease the messiness of the classroom. “Don’t eat in the class”, she tells Alberto and Gaspar, who keep eating once she turns around. Everybody is talking and playing. After making a gesture of exasperation, Rosario positions herself in front of the class, facing the students. She then commands them to move their desks in order to create more distance between them, apparently hoping that distance would diminish their eagerness to talk. Her manoeuvre only partially works.

As a further effort, Rosario calls Pedro to the front of the classroom. He is a 10-year old boy, who was diagnosed with ADHD a couple of years ago. After taking medication for over a year, his parents and treating psychiatrist decided to stop the pharmaceutical treatment this academic year. However, after one and a half months without medication, Pedro’s parents, teachers and the psychiatrist supervising his case decided that the best thing was to go back to the pharmaceutical treatment. Pedro agreed to this decision, arguing that he was having trouble keeping up with his classmates. During an interview he mentioned that staying focused was something that slipped out of his control, making him feel frustrated during class. “I feel like I don’t understand anything, so I get bad grades, so I stop even trying to pay attention. Because, what’s the point? I’m gonna fail anyway, so I rather have fun”, he told me once while we were chatting during the break. Since he again started his pharmaceutical treatment 1 week ago, teachers constantly try to help him keep interested in the class by pushing him to contribute in several activities.

“You’re going to be my assistant today,” Rosario tells Pedro. “Your duty is to write the name of anyone who is making a mess.” Pedro nods, grabs a magic marker, and starts surveilling the class. He executes this role playfully. He smiles at his classmates and quietly jokes with them but, also, he starts listing some names in the whiteboard. Because he kept eating despite having already been told to stop, Pedro writes Alberto’s name on the whiteboard. Alberto shouts at him, saying that he was not doing anything wrong. But Pedro stays firm with his decision. He seems focused on playing his role properly. Alberto shouts to Pedro “have you had your medication today?” in an attempt to mock him, although Pedro does not seem to mind the comment at all. Alberto then stands up, and goes where Pedro is standing. “Please, erase my name from the list. I didn’t do anything wrong,” he says. “You were eating, and that is not allowed,” Pedro replies. Alberto heads back to his seat. He seems slightly annoyed, but after a few minutes he does not seem to care anymore. After a while, Rosario tells Pedro to go back to his desk and to start working on that day’s assignment. As time passes and children gradually become focused on their work, Rosario comes to where I am standing and whispers to me to look at Pedro. He is not working on the activities as instructed—instead, he is looking out the window, while softly chewing a pair of scissors. In response to my request for her to explain what she made of the situation, she replied: “you need to keep them interested, you need to get them involved. All children, but especially the ones with learning difficulties.”

“Keeping them interested” is a catchphrase that is regularly used by Rosario and her colleagues, when describing efforts to achieve a “productive learning environment”. Although described with slight differences, most teachers shared the idea that in order for children to really learn, their attention should be aimed at the content, and so should their interest. Every little thing in the classroom should be direction to this goal, every material object, every intervention performed by the teacher, and the interactions between the children. All of these are supposed to contribute, to be subsidiary to the final goal of learning.

From its early beginning, this educational institution has made particular ethical and pedagogical considerations that are aligned with Catholic principles. Decisions made for the sake of providing a productive learning environment are deeply influenced by Catholic values. For example, one such decision involved organizing the educational institution into two schools—Mount Sinai and Bethlehem—in order to keep boys and girls in separated classrooms. But these traditional Catholic values do not just translate into anecdotal things such as the segregated spaces. They directly impact in institutional practices related to how children should behave, what skills and values they should develop, and therefore tension the classroom setting in terms of how and why teachers can intervene, and they provide a baseline of how children should behave. According to staff members, the Catholic values are reflected in institutional practices and codes of conduct. For instance, children are expected to develop from an early age the capacity to self-regulate their impulses, acting accordingly to what is required in every occasion according to Catholic values. This means that children should respect and live according to their individual originality, but at the same time they must “educate their liberty”—as the school’s project states—towards the search of truth and goodness. This is why discipline is ideally not imposed by an external actor upon the child in these schools. Teachers are allowed to nag children, and to perform different disciplinary measures. However, this is discouraged under the expectation that children will act properly. They are expected to realize what is right and wrong by themselves, as the result of a process of personal insight that should happen early during life, and continue during their lifespan.

But the expectations that the educational institution has regarding how children should act and behave are constantly tensioned in practice. As it is revealed in the story previously shared, teachers execute different manoeuvres and techniques attempting to help guide the child’s interest towards the academic content. Despite the educational institution’s expectation that children can and should self-regulate, this skill appears to be in need of constant external aid. It is possible then to witness an interplay between external elements aiming at aiding the child to develop certain skills, and the ways that in practice these interventions performed by teachers and other adults are received by the child in the everyday interactions in which children are involved. Stimulant medication plays a central role in how these dynamics unfold, and the outcomes they have. Psychostimulants are a core ingredient in the establishment of particular kind on entanglement produced in the classroom—-a pharmaceutical entanglement—and they are also key to understanding how through these entanglements agency can be strengthened, produced or dispelled.

During an English class that took place a few days after the events listed above, the teacher, in an attempt to keep in control of the class, decided that she needed an assistant to list down the names of those playing and talking, hence paying little attention to what she was saying, or that were disrupting other children who were trying to focus on the class. So she calls Nicolás to the whiteboard. Also diagnosed with ADHD, Nicolás has already been under medication for 2 years. He starts gazing at the classroom, attentive to even the minimal amount of noise or movement, and every time he glimpses something, he does not hesitate to list a name on the whiteboard. In a few minutes, no more than three, seven names have already been listed down. Nicolás seems proud of the way he is handling his assignment. While normally he is being teased by his classmates because he is funny looking, and acts a little more childish than most of the class, now he is in a position where he can dictate what is happening in the room. His eagerness to pay attention to the minimal detail of what is happening around him at the moment contrast with his day-to-day behaviour. On a daily basis, and despite having the medication, he is normally easily distracted by random stimuli. Additionally, he is one of those children who have a difficult time when asked to remain silent. Whether it is to comment on the topics of the class—which he sometimes adequately does, but most of the times he does not—drifting away to topics of his own interest, or just to chat about anything, Nicolás seems to constantly have difficulties to connect to what is happening inside the classroom and to restrain himself from acting back to whatever happens around him. But now, right in this moment and in this context where he can strike back at those who normally play him for a fool, he seems focused as never before. But his will to excel in his new-found position backfires when the teacher realizes that almost 15 names are listed on the whiteboard in less than 10 minutes. “Go back to your seat Nicolás, you’re not taking this seriously” she says. Nicolás argues that he was only doing what he was told. “No, you weren’t, you’re just joking around. Go and sit, and pay attention to what has been explained so far in the class”. Nicolás looks baffled, while the rest of the class burst into laughter.

Not long after, another child diagnosed with ADHD caught my attention. Gabriel has been under pharmacological treatment for over a year. However, since I met him for the first time, he never impressed me as a child whose characteristics and personality traits have been overwritten as a consequence of having the medication. Although rather mischievous in his actions in the classroom, he tends to act accordingly to what is expected from children in such setting. From time to time he does something that can be considered as out of line, but the rest of the time he keeps up the pace demanded by the teachers. On this particular day, he exhibits some traits of the behaviour that made his parents ask for assistance from a medical expert in the first place. While Nicolás is listing names in the whiteboard, Gabriel is sitting down. He splits his time between paying attention to what is being said by the teacher, and chatting quietly with the boy sitting next to him. But once Nicolás is sent back to his desk, Gabriel stands up and walks towards the garbage can. As he walks there through the back of the room, he kicks the chair where other boy is sitting, making him fall down. Gabriel laughs. And so do the ones that saw what happened. Gabriel keeps walking as if nothing has happened. He jokes around, blows his nose, throws the paper tissue away and comes back to his seat. All of this in less than two minutes.

For the following 30–45 minutes, Gabriel combines moments of working, where he seems able to focus and be responsive to what the teacher is saying, with others of recreation. But also, he seems to be able to mix both together. As Alejandra asks Gabriel some questions about the proper pronunciation of some English words, Gabriel replies theatrically, exaggerating the intonation of the words, making them sound absurd. The teacher smiles at Gabriel, and he smiles back. Alejandra seems pleased with what just happened, as if it did not matter that he made a joke out of being examined, as long as he was able to reply. “That reveals that he was focused, paying attention on what was being said,” she will later confess to me. For his part, Gabriel turns around and starts chatting with another boy. “I just love teasing the teacher,” I heard him say.

These stories illustrate the nuanced and complex ways in which pharmaceutical entanglements take place. In them, it is possible to observe how the effects of the medication can produce something different than the commonly caricatured images of medicated children, such as those pushed into severely numbed states, or those who are dramatically transformed into model students after beginning medications.

The stories also make apparent how the encounter between child and medication takes place in the midst of other, different interactions that also influence how medication effects unfold. Classmates, teachers, pedagogical techniques, whiteboards, scissors and magic markers, these and more become intertwined in actual classroom settings, the particular sociomaterial context in which pharmaceutical entanglements take place. In this sense, it is possible to observe that the presence of psychostimulants in the classroom is better described as the introduction of a potentiality. Thinking about psychostimulants as a potentiality entails that a highly dynamic and open-ended process must take place, a process that might lead a child to become different (Taussig, Hoeyer, & Helmreich, 2013). However, how this difference, introduced by the use of stimulant medication in the classroom, unfolds is open to different outcomes, which are dependent and rely on how stimulant medication becomes entangled with the other actors, particularly with children using it. Considering this, we discern two potential pharmaceutical entanglements concerning children and medication. One that can be described as a gentle and nuanced coming together of both actors—medication and the child—into what can be considered as a proper fit. When this happens, stimulant medication serves to foster the emergence of certain qualities upon the child—qualities that are considered as desirable and useful by the child and others that compose his or her environment. The other possible entanglement reported by teachers and students corresponds to when the medication imposes itself upon what is considered as the previous version of the child. When this happens, the medication is considered to be a threat because of the potentiality it carries to modify the child to such extent that it becomes difficult to recognize some of his or her previous characteristics. In terms of the effects it entails on the medicated child’s subjectivity, we claim stimulant medication is neither strictly beneficial nor harmful by itself.

Colomba, a 10-year-old girl who participated in the research, makes an insightful statement in relation to the different paths that medication can undergo. Using a scene from a Harry Potter’s film, she reflects upon the potential effects that the entanglement between a child and medication can have:

I think that, for instance … ok, I think that the medication that you have for improving your mood, or for focusing or any of those things … they will be good for you if you want to have them in order to be able to do something. But I sort of have the feeling that, I don’t know, that they’re vitamins that they just put in a jar. And they say to you “ok, if you have them, this will help you focus more”. And you have the medication because you’re forced to, and it doesn’t work. But if you want to take it because you want to focus more, you’ll focus more, and you’ll think the medication helped you to do so. In a movie, in Harry Potter, this guy has to go audition to become a quidditch goalkeeper. Well, the thing is that Harry gives him some drops that supposedly bring good luck. And this guy believes that the drops work, and he wins. And Hermione asks Harry “did you put the magical drops?”, and Harry replies “I only faked that I did it”. But since Ron thinks that he drank the magic drops, he plays as a goalie and he’s actually good at it. And he wins. But the reality is that the drops didn’t affect him at all, but thanks to them he’s able to do it (to succeed).

Colomba’s reflection bolsters our own impressions of how these pharmaceutical entanglements take place. She emphasizes two things. First, that medication is a constraint. It is not something wanted by most children. But also, she highlights that there might be a benefit to be derived from such constraint. In reality, medication is neither water, nor magical drops. Although commonly reflected by children as lacking attributes powerful enough to force change, psychostimulants produce something upon the body. It is not neutral, but this does not mean that it cannot be beneficial.

The use of stimulant medication introduces a constraint that was not previously experienced by the child. However, constraints are not to be deemed only in a negative, restrictive fashion, as it is revealed in the daily dynamics registered in the stories we shared. Gomart (2002, 2004) discusses a similar matter when reflecting upon the role that drugs may have upon those taking them. She realizes that most accounts start under the assumption that the individual is either an active and rational being, or that he is controlled and coerced by the drugs. Gomart wonders why is it that social sciences have considered the individual as a “close entity”, already formed and complete. In this belief she pinpoints the main reason why drugs are considered to corrupt the individual. Since actors are normally considered “(as entering) the scene as already formed and filled to the brim with capacities, intentions and desires, ‘Action’, then is the expression of these inherent properties: for this manifestation to be complete, the entities must be the only actors on stage: if others act at the same time, this manifestation is corrupted” (2002, p. 520).

Put another way: what if the individual is not corrupted by other actors, but is rather enabled by them, granted—due to other actors—of certain capacities? For Gomart, the answer is simple: entities, such as the medicated child, are not the result of merely human action, nor of the actions forced upon them by objects. The individual is “the result of practices that frame, embody, localize and temporize” (2002, p. 520). By acknowledging this, the focus of analysis shifts from trying to eliminate constraints in order to make the individual autonomous and free, to distinguish what kind of forces, what constraints may act positively, inducing movement. The effects of the medication can be forced upon the child—normally reported as negative effect—but they can also play along the child’s interest in acting differently. In this case, the effects of the medication do not just happen. They have to be performed by active agents (Gomart, 2004). And agents can also be guided into becoming active, and Pedro makes a fair example of this. Although all children are normally exposed to constant activities in order to keep them interested and focused during the class, attempts to accomplish this are redoubled with medicated children. Additionally, teachers are more attentive to the effects such actions have when they are aimed at medicated children. Once Pedro started having medication, teachers tried by different means to capture his attention, to keep him focused and active during the class.

As mentioned, it is not enough to simply have the medication in order for something to happen. Introducing such an actor in the array of arrangements performed in the classroom makes it necessary for the medicated child to acknowledge the presence of a new constraint, a modification in the choreographic dance that has previously been enacted. But this constraint can work as more than just a limitation. Constraints such as stimulant medication can be generous, they can modify the set of arrangements, and they can induce movement, producing a new choreography (Cussins, 1996; Thompson, 2005). But in order for this to happen, its agency has to converge with that exhibited by the medicated child. It is a very subtle interrelation that takes place. If the medication overcomes the medicated child, and he or she shows no sign of working along the medication, then its effects are null or adverse. However, if actions induced by the medication are joined by an active agent trying to perform the effects of the medication, a space for novelty opens.

In practice, medicated children are normally guided by their teachers or even their peers in order for the medication to produce its beneficial effects. This guidance works as an external control locus for the medicated children who, despite having the medication, reveal little interest in focusing in the class. This is particularly relevant since that whether they choose to work with the medication or not, it appears that normally there is always room for children’s actions to modify what is happening in the classroom. Rarely the medication revealed itself as imposing itself over the child. Normally, children having the medication conducted themselves in the same way that other children do: they fool around, play and talk with their peers. Only a few children appeared to be negatively constrained by psychostimulants, as when a student would seem unwilling to leave their desk, or would behave towards others in a way that was perceived as unusual or inappropriate. Normally, the changes related to the introduction of the medication are subtle, and bursts of previous ways of behaving are always on the verge of taking place.

## The potentialities of interacting agents

In a classical essay regarding how individuals become regular marihuana users, Becker (1953) argued that drug experiences involve more than the chemicals and biology that were the focus of laboratory drug research. Observing how marijuana is used in everyday settings, he became convinced of the inadequacy of scientific accounts which focused on drugs’ biological modes of action, and the effects of these as perceived by users. The experience of ‘being high’ was not something that could be produced by drugs alone, but was in fact contingent upon these being interpreted in particular ways that were learned through social processes. Thus, Becker argued that “becoming a marijuana user’ could not happen without social interactions that provided the specific meanings and expectations needed for interpreting marijuana use as an enjoyable experience, worthy of repeating—an insight that has been supported by the findings of a variety of other researchers, including those involved in clinical and laboratory-based studies (Fraser, 2011; Robins, Davis, & Nurco, 1974; Zinberg, 1984).

Similarly, the interaction between the child and the medication requires some actions to be taken in order to fit together in such a way that it is felt as advantageous for the child. First, children must learn how to perceive and interpret the effects of psychostimulants. The medication produces signals that the child must learn to decode, to recognize and use in order for something new to emerge. The action of taking the medication cannot be reduced to a cause–effect relationship where the medication triggers a novel ability, or crumbles a former self. In this sense, stimulant medication operates as a potentiality, which can be deployed in various ways that yield a range of different outcomes—from the coercive control of children, to the increased self-control of children, and the reshaping of student subjectivities (Rose, 2006). And secondly, these bodily and mental sensations must be recognized as purposeful. That is, they must be associated with a purpose that renders sensible the fact of having the medication in the first place. Otherwise, the same bodily sensation can be felt as disruptive, annoying, or threatening.

Emotional elements also play a role in how the actual experiences, practices and meanings arising from the use of psychostimulant medication take place (Vrecko, 2013). Drawing on his fieldwork experience with university students, Vrecko concludes that changes in the emotional states of students using methylphenidate are central for understanding how they shape their perceptions of how the interactions with the medication occur. Among the different findings he describes, one proves to be particular enlightening for our argument: There is a certain feeling of drivenness that comes with the ingestion of methylphenidate. But this drivenness, which is described as “feeling a strong need or desire to do something” (p. 6) must be channelled. It does not come with a pre-fixed goal, but instead must be directed somehow, in order to achieve a specific goal.

If the potentialities of the medication are not exclusively contained in the pill itself, where are they to be found? The answer appears to lie in how new modes of agency can emerge in the interactions that both the medication and the medicated child develop as the result of their mutual interconnections, and as the result of their own multiple understandings. In the Gabriel and Nicolás cases it is possible to witness how stimulant medication can foster their capacity to focus. However, how they make use of this increased capacity differs according to their own characteristics. While Nicolás makes what is considered by his teacher as a poor use of such ability, guided more by his desire to playfully take revenge on his classmates, Gabriel seems more skilfully to switch from accomplishing the teacher requirements to goofing around. In practice, what stimulant medication allows them to achieve is not linked to a pre-fixed ideal of school performance. The agency produced as the outcome of this pharmaceutical entanglement has to be put into action by the child. A “model student” and a medicated child whose entanglement with the medication can be considered according to his own experience as a “virtuous” one,^1^ are not overlapping categories. In this sense, stimulant medication can also unfold and be linked to attempts conducted by the medicated child to do things which serve their own interest, and not only to fit what is expected out of them in the classroom. Gabriel serves as a fair example of this. The way in he makes use of the medication allows him to fulfil partially what is expected of him in terms of academic conduct, and since he can put up this façade, he can manage to do the other things he wants to do, such as teasing the teacher, or joking around. But apparently he only can get away with this because he is having the medication. This way, it is possible to talk about an “agency of the medication” and about an “agency of the medicated child”. Although these can be considered as different and independent phenomena, in practice both appear to be intertwined together.

On the one hand, the "agency of medications" corresponds to the new set of arrangements, complicities and entanglements that emerge in the classroom because stimulant medication appears as a new actor in the scene. As mentioned, children under medication are to some extent addressed differently, they are guided into action by their teachers, they are constantly being examined but they are also constantly being given second chances: more time, more space, and more room to “be” and to dwell in the classroom. On the other hand, the “agency of medicated children” corresponds to how, after learning to recognize and master the bodily sensation that come along the ingestion of the medication, a child can make use of the medication in order to achieve new things, to interact with those who compose the classroom environment differently, accomplishing things that are considered meaningful by them. As mentioned, neither of these types of agency necessarily translate into better school performance if not guided towards that direction by the child, which may need help in doing so from their teachers or peers.

## Conclusion

The conditions of the medicated children appearing in the stories above—and those in the school classrooms we observed—bear little resemblance to the numbed states described by those who oppose pharmacological treatments under the argument of a potential collapse of the child’s subjectivity (i.e. Breggin, 2001). We are not suggesting that such states do not happen. Rather, we are suggesting that they represent only some of the many potential states that may arise in relation to medication use; and that they were not frequently observed or reported within the particular settings of our fieldwork.

In accordance with the above discussion, we claim  that while medicalization theories  may offer conceptual tools that are helpful for understanding some of the general dynamics associated with the use of psychopharmaceuticals to manage deviant behaviour, they do not provide a framework that is adequate for understanding the multiple contextual determinants involved in how the medication unfolds in practice in children’s everyday lives. As this research shows, ethnographic accounts can challenge the assumptions that pharmaceutical drugs act only in terms of coercion and control. Children who took part in this study often seem to make use of the medication in manifold ways. In order to understand how the effects of stimulant medication unfold in the classroom, it becomes crucial to attend to the role played by sociomaterial configurations.

What we are suggesting is that the states and experiences associated with medication use are, to a significant extent, a product of the interactions between the medicated child and the stimulant medication. As mentioned earlier, this process of mutual interaction seems to induce modification in the ways the medicated children behave when the medication manages to match the children’s interests into acting differently. This way, stimulant medication seems to work along with their interest in achieving certain goals. But this interaction also modifies the medication itself. If there was something such as a pre-fixed idea of what the medication can accomplish, of how it should unfold once the child ingests it, this expectation might need to be re-examined under a different scope. It is not only what methylphenidate is expected to do, and it is not only about the reported perils linked to its misuses, abuse or side-effects. What the different outcomes of this interaction reveal to us is that these potential effects do not necessarily just happen, triggered by the mere act of having the medication. These unfoldings are also put into motion depending on how the interaction between stimulant medication and the child takes place. In this sense, both parties play a highly influential role on what can be considered as the overall result, the medicated child as he or she presents him/herself in the classroom.

The medicated child seems to be more than just a simple addition of the child’s previous identity traits, plus the expected effects that the medication can induce (these being either the ones considered as beneficial, or the negative and unexpected side-effects). Instead of a simple addition, what happens is a recurrent interplay between the child and the stimulant medication, where they constantly relocate and make use of each other in different ways. In this constant process of mutual re-accommodation, the medicated child can learn how to make use of the potentiality induced by the psychostimulants, can dismiss it, or can even fight against it. All of these potential destinations can be achieved within different ranges. We suggest that in order to measure the quality of these arrangements it is better not to think of them in terms of simple good or bad entanglements, since medicated children normally do not perform these in absolute terms. As exemplified in our account of Gabriel’s story, a child can work with medication effects in different ways, achieving different outcomes in different situations. At times, Gabriel’s medication appears to help him behave “mischievously” (according to his own words, and to what his teacher said to the researcher), while at other times, it seems to help him become the sort of engaged and active learner that is expected of children in classroom settings, which he often struggles to do when not taking medications.

Ultimately, this suggests that pharmaceutical entanglements between the child and medication may be enacted in different ways, which alter how the effects of medications come to be manifested in the classroom settings. Stimulant medications are themselves important factors that alter the experiences of children and dynamics of classrooms, but they do not work on their own, and do more than simply constrain what children can do. From such a perspective, it becomes possible to consider children on medication as more than just the passive subjects of medical discipline, controlled by the pharmaceutical regimes imposed upon them. It becomes possible to see children as active (though by no means free) agents that work alongside other elements of the sociomaterial configurations that together produce particular drug effects. While this does not necessarily make it easier to come to simple conclusions about whether the use of drug treatments for ADHD-diagnosed children ought to accepted or condemned in general terms, we believe it does allow for a more accurate understanding of the complexities of how such drugs actually work—in many potentially different ways, which are determined not just by the properties of drugs, but through a set of sociomaterial entanglements and contingent relations that demand careful analytic attention.

## Note

1. By a “virtuous” entanglement we refer to an entanglement that is felt as beneficial by the child since it endorses characteristics which are felt as positive by him or her, or it allows the emergence of capacities that are experienced as beneficial by the child.
